# Influence of self-efficacy and self-directed learning competence on core competence among nurses specializing in infectious diseases: a cross-sectional study

**DOI:** 10.3389/fpubh.2026.1775251

**Published:** 2026-05-22

**Authors:** Jian-Wei Zhang, Li-Hong Yue, Yan Wang, Jia-Yu Duan, Hai-Hong Shan

**Affiliations:** 1 Department of Nursing, Shanxi Bethune Hospital, Shanxi Academy of Medical Sciences, Tongji Shanxi Hospital, Third Hospital of Shanxi Medical University, Taiyuan, Shanxi Province, China; 2 Department of Infection, Shanxi Bethune Hospital, Shanxi Academy of Medical Sciences, Tongji Shanxi Hospital, Third Hospital of Shanxi Medical University, Taiyuan, Shanxi Province, China

**Keywords:** core competence, infectious diseases, nurses, self-directed learning competence, self-efficacy

## Abstract

**Objective:**

This study aimed to assess the current level of core competence among nurses working in infectious disease settings and to examine the influence of self-efficacy and self-directed learning competence on core competence.

**Methods:**

A total of 392 nurses specializing in infectious diseases were selected through convenience sampling. Data were collected using four instruments: a General Information Questionnaire, the Core Competence Scale for Infectious Disease Specialist Nurses, the Self-directed Learning Competence Scale for Nurses, and the General Self-efficacy Scale.

**Results:**

The mean core competence score among participants was 133.72 ± 24.06. The mean score for self-directed learning competence was 134.72 ± 25.35, and the mean score for self-efficacy was 30.17 ± 6.78. Core competence scores were positively correlated with both self-directed learning competence and self-efficacy. Multiple regression analysis identified hospital grade, job position, years of work experience, teaching experience, self-directed learning competence, and self-efficacy as significant factors associated with core competence (*p* < 0.05).

**Conclusion:**

Nurses engaged in infectious disease care demonstrated above-average core competence. Self-directed learning competence and self-efficacy were identified as primary influencing factors. Interventions aimed at enhancing these competencies may support nursing managers in strengthening workforce capacity in infectious disease care.

## 1 Introduction

Nurses specializing in infectious diseases play a critical role in delivering clinical care to patients and supporting efforts in the prevention and control of infectious diseases. In recent years, the field has been shaped by both significant challenges and emerging. The increasing incidence of emerging and re-emerging infectious diseases, such as COVID-19 and other novel pathogens, has placed substantial demands on healthcare systems and frontline nursing staff (1). Advances in medical technology, infection control strategies, and professional training systems have created new opportunities to enhance nursing practice and improve patient outcomes (2).

Core competence encompasses the set of knowledge, skills, and professional qualities required for effective clinical nursing practice (3). The concept of core competence was first introduced in nursing research in the United States and was later adapted by Chinese scholars to evaluate the competence of specialist nurses (4–6). Among infectious disease nurses, the core competence is closely associated with the quality of patient care and holds considerable importance for public health outcomes. Globally, research on nursing core competence has gradually shifted from general clinical competence to more specialized domains, including infection prevention and control, emergency response capacity, and interdisciplinary collaboration (7). Studies conducted in developed healthcare systems have highlighted the importance of continuous professional development in improving clinical performance and patient safety (8). In addition, increasing attention has been paid to psychological and behavioral factors, such as self-efficacy and self-directed learning ability, which are considered key determinants of professional competence and lifelong learning among nurses (9). Increasing evidence suggests that higher levels of self-efficacy are associated with improved clinical decision-making, adaptability, and resilience under high-pressure conditions, while self-directed learning ability plays a critical role in maintaining up-to-date knowledge and skills in rapidly evolving clinical environments (10). In China, several studies have examined the competence level of infectious disease specialist nurses and associated influencing factors (11). However, prior investigations predominantly relied on observer-rated questionnaires, and included a limited number of variables, which constrained the depth of analysis regarding interrelationships among influencing factors. Some investigations have explored associations between core competence, self-efficacy, and self-directed learning competence among clinical nurses (12). However, research specifically focusing on infectious disease nurses remains limited.

Given the unique competence requirements of infectious disease nurses, including expertise in infection prevention and control, and the ability to respond to epidemic outbreaks, the present study aimed to assess core competence levels in this population and to evaluate the impact of self-efficacy and self-directed learning competence. These findings aim to provide a scientific basis for developing targeted strategies to enhance core competence among nurses in infectious disease settings.

## 2 Participants and methods

### 2.1 Participants

Between September and November 2024, nurses engaged in infectious disease care were selected from 10 general and specialized hospitals in North China, Central China, and South China through a convenience sampling method. Specifically, the project leader distributed the questionnaire to known contacts who were responsible for infectious disease departments in selected hospitals. These contacts then assisted in forwarding the questionnaire to eligible nurses within their departments for voluntary participation. Most of these included hospitals were tertiary (Grade III) general hospitals or specialized hospitals.

Inclusion criteria were: possession of a valid nurse qualification certificate, a minimum of 1 year of experience in infectious disease nursing, and voluntarily participation with provision of informed consent.

Exclusion criteria were: nurses who were on leave (maternity, sick, or personal) during the investigation period; participation in off-site training programs; and status as a trainee or student nurse.

This study included 13 general demographic variables, 5 dimensions from the Infectious Disease Specialist Nurse's Core Competence Scale, 4 dimensions from the Scale of Self-directed Learning Competence for Nurses, and 10 variables from the General Self-efficacy Scale. The sample size was estimated based on the rule of at least 10 participants per variable, with an additional 10% to account for potential invalid responses, resulting in a required sample size of 352 (13). To further ensure adequacy, the target sample size was increased to 387. Ultimately, a total of 392 questionnaires were collected. Ethical approval for this study was granted by the Ethics Committee of Shanxi Bethune Hospital (YXLL-2024-204).

### 2.2 Instruments

#### 2.2.1 General information questionnaire

This investigator-designed questionnaire collected demographic and professional background information, including hospital grade, hospital type, age, marital status, fertility status, educational background, professional title, job position, employment status, years of professional experience, teaching experience, participation in continuing education, and specialist nurse status.

#### 2.2.2 Infectious disease specialist nurse's core competence scale

Developed by Wu et al., this scale consists of 34 items across 5 dimensions (11): professional development competence (11 items), infection prevention and control capacity (9 items), infectious disease nursing capacity (6 items), professional humanistic quality (5 items), and infectious disease epidemic response capacity (3 items). Items are rated on a 5-point Likert scale ranging from 1 (“very inconsistent”) to 5 (“very consistent”), yielding a total score ranging from 34 to 170. Higher scores reflect higher levels of core competence. Psychometric properties on the scale include a Cronbach's α coefficient of 0.806, test-retest reliability of 0.831, and content validity of 0.869.

#### 2.2.3 Scale of self-directed learning competence for nurses

Developed by Xiao, this scale includes 34 items across 4 dimensions: self-motivation belief (14 items), task analysis (6 items), self-monitoring and regulation (10 items), and self-evaluation (4 items) (14). Items are rated on a 5-point Likert scale ranging from 1 (“completely inconsistent”) to 5 (“completely consistent”), with total scores ranging from 34 to 170. Higher scores indicate greater self-directed learning competence. The Cronbach's α coefficient is 0.944.

#### 2.2.4 General self-efficacy scale

Compiled by Schwarzer et al., this scale comprises 10 items, each rated on a 4-point Likert scale from 1 (“completely incorrect”) to 4 (“completely correct”) (15). Total scores range from 10 to 40, with higher scores indicating stronger self-efficacy. The Cronbach's α coefficient is 0.87.

The complete version of the questionnaire is shown in Supplementary Material.

### 2.3 Data collection methods

Data collection was carried out using the Wenjuanxing platform integrated within WeChat. The study's objective and questionnaire procedures were communicated by the project leader to the department head or the head nurse of the infectious diseases unit. The questionnaire link was distributed to eligible nurses. Standardized instructions were provided within the questionnaire, and participants accessed the link and completed the questionnaire after providing informed consent electronically. Completion time ranged from approximately 15 to 20 min. A total of 399 questionnaires were collected; after excluding 7 containing logical errors, 392 valid responses were retained. The effective response rate was 98.24%.

### 2.4 Statistical analysis

Data were analyzed using SPSS version 26.0 (IBM Corp., Armonk, NY, USA). Univariate analyses were conducted using independent-samples *t* tests or one-way analysis of variance. Pearson's correlation coefficients were calculated to examine associations among variables. Multiple linear regression analysis was conducted to identify factors influencing core competence. A *p* value < 0.05 indicated statistical significance.

## 3 Results

### 3.1 Scores of core competence, self-directed learning competence, and self-efficacy among infectious disease nurses

The scores of core competence, self-directed learning competence, and self-efficacy among infectious disease nurses are presented in Table 1. Among the five dimensions of core competence, professional humanistic quality demonstrated the highest score, whereas professional development competence demonstrated the lowest score. Within the four dimensions of self-directed learning competence, self-motivation belief demonstrated the highest score, and self-evaluation demonstrated the lowest score.

**Table 1:** Scores of core competence, self-directed learning competence, and general self-efficacy among infectious disease nurses (*n* = 392, $\bar{x}$ ± *s*).

Item	Total score	Mean item score
Total score of core competence	133.72 ± 24.06	3.93 ± 0.71
Professional development competence	37.76 ± 10.57	3.43 ± 0.96
Infection prevention and control capacity	38.10 ± 6.79	4.23 ± 0.75
Infectious disease nursing competence	23.89 ± 4.60	3.98 ± 0.77
Professional humanistic quality	21.72 ± 3.54	4.34 ± 0.71
Infectious disease epidemic response capacity	12.24 ± 2.35	4.08 ± 0.78
Total score of self-directed learning competence	134.72 ± 25.35	3.96 ± 0.75
Self-motivation belief	56.28 ± 10.54	4.02 ± 0.82
Task analysis	23.52 ± 4.90	3.91 ± 0.79
Self-monitoring and regulation	39.52 ± 7.85	3.95 ± 0.79
Self-evaluation	15.41 ± 3.04	3.85 ± 0.76
Total score of general self-efficacy	30.17 ± 6.78	3.02 ± 0.68

### 3.2 Differences in core competence scores by participant characteristics

Univariate analyses indicated significant differences in core competence scores across hospital grade, age, marital status, fertility status, professional title, job position, employment type, years of clinical experience, teaching experience, further education experience, and specialist nurse qualification (*p* < 0.05). Detailed results are presented in Table 2.

**Table 2:** Core competence scores among infectious disease nurses by demographic and professional characteristics (*n* = 392).

Item	Number of nurses [*n* (%)]	Total score (point, $\bar{x}$ ±*s*)	F/t	*p*
Hospital grade			4.515	0.004
Grade A secondary	63 (16.07)	124.97 ± 21.01		
Grade B secondary	6 (1.53)	129.00 ± 15.30		
Grade A tertiary	321 (81.89)	135.34 ± 24.37		
Grade B tertiary	2 (0.51)	164.00 ± 8.49		
Hospital category			2.218	0.137
General hospital	228 (58.16)	135.25 ± 23.04		
Specialized hospital	164 (41.84)	131.59 ± 25.33		
Age			5.055	0.002
21–30 years	125 (31.89)	127.82 ± 24.41		
31–40 years	192 (48.98)	134.73 ± 24.01		
41–50 years	61 (15.56)	141.03 ± 21.61		
>51 years	14 (3.57)	140.79 ± 21.29		
Marital status			9.414	< 0.001
Unmarried	101 (25.77)	125.52 ± 22.52		
Married	288 (73.43)	136.35 ± 23.95		
Other	3 (0.77)	157.33 ± 17.01		
Fertility status			4.712	0.010
None	140 (35.71)	128.76 ± 24.48		
1	169 (43.11)	136.53 ± 22.77		
≥2 children	83 (21.17)	136.36 ± 24.84		
Education level			2.435	0.064
Technical secondary school	3 (0.77)	128.33 ± 16.01		
Junior college	26 (6.63)	133.96 ± 23.00		
Undergraduate	353 (90.05)	133.18 ± 24.20		
Master's degree or above	10 (2.55)	153.70 ± 15.83		
Professional title			5.902	< 0.001
Nurse	71 (18.11)	126.54 ± 23.97		
Nurse practitioner	158 (40.31)	131.05 ± 24.46		
Nurse-in-charge	141 (35.97)	137.78 ± 22.76		
Associate chief nurse	16 (4.08)	150.31 ± 17.37		
Chief nurse	6 (1.53)	149.50 ± 20.84		
Job position			5.399	0.001
Clinical nurse	359 (91.58)	132.29 ± 24.07		
Nursing teaching position	6 (1.53)	148.83 ± 19.97		
Nursing management position	23 (5.87)	148.09 ± 18.30		
Nursing scientific research position	4 (1.02)	157.25 ± 12.69		
Employment nature			9.509	< 0.001
Contract-based	315 (80.36)	131.78 ± 24.10		
Public institution	67 (17.09)	144.61 ± 21.77		
Other	10 (2.55)	121.80 ± 15.53		
Years of work experience			4.966	< 0.001
≤ 2 years	57 (14.54)	125.81 ± 28.07		
3–5 years	79 (20.15)	132.43 ± 22.07		
Hospital grade		
6–10 years	77 (19.64)	130.13 ± 24.64		
11–15 years	100 (25.51)	135.03 ± 23.49		
Above 15 years	79 (20.15)	142.57 ± 20.46		
Teaching experience			23.943	< 0.001
Yes	220 (56.12)	138.83 ± 20.45		
No	172 (43.88)	127.19 ± 26.68		
Further education experience			10.110	0.002
Yes	118 (30.10)	139.54 ± 22.58		
No	274 (69.90)	131.22 ± 24.28		
Specialist nurse certification			5.693	0.018
Yes	136 (34.69)	137.68 ± 23.90		
No	256 (65.31)	131.62 ± 23.93		

### 3.3 Correlation between core competence, self-directed learning competence and self-efficacy

Pearson's correlation analysis indicated that the total core competence scores was positively correlated with both self-directed learning competence and self-efficacy (*p* < 0.001). These findings are presented in Table 3.

**Table 3:** Correlations among core competence, self-directed learning competence, and general self-efficacy among infectious disease nurses (*n* = 392).

Item	Total nurses' self-directed learning competence score	General self-efficacy	Self-motivation belief	Task analysis	Self-monitoring and regulation	Self-evaluation
Total core competence score	0.795^**^	0.663^**^	0.811^**^	0.736^**^	0.755^**^	0.677^**^
Professional development competence	0.635^**^	0.572^**^	0.633^**^	0.610^*^	0.608^**^	0.541^**^
Infection prevention and control capacity	0.691^**^	0.555^**^	0.724^**^	0.618^**^	0.653^**^	0.572^**^
Infectious disease nursing competence	0.750^**^	0.632^**^	0.757^**^	0.696^**^	0.715^**^	0.663^**^
Professional humanistic quality	0.707^**^	0.510^**^	0.748^**^	0.624^**^	0.660^**^	0.594^**^
Infectious disease epidemic response capacity	0.751^**^	0.610^**^	0.754^**^	0.703^**^	0.719^**^	0.659^**^

^**^represents *p* < 0.001.

### 3.4 Multivariate analysis of factors influencing core competence

A multivariate linear regression analysis was conducted to identify factors associated with core competence. The total core competence score was entered as the dependent variable. Independent variables included those found to be significant in the univariate analysis, along with the total scores of self-directed learning competence and self-efficacy. Variable coding details are presented in Table 4. Regression analysis results indicated that hospital grade, job position, years of work experience, teaching experience, self-directed learning competence, and self-efficacy were significant influencing factors of core competence among infectious disease nurses (*p* < 0.05), as presented in Table 5.

**Table 4:** Coding of independent variables for multivariate linear regression analysis.

Variable	Assignment
Hospital grade	1 = Grade A secondary; 2 = Grade B secondary; 3 = Grade A tertiary; 4 = Grade B tertiary
Age group	1 = 21–30 years old; 2 = 31–40 years old; 3 = 41–50 years old; 4 = >51 years old
Marital status	1 = Unmarried; 2 = Married; 3 = Other
Fertility status	1 = 0; 2 = 1; 3 = 2; and above
Professional title	1 = Nurse; 2 = Nurse practitioner; 3 = Nurse-in-charge; 4 = Associate chief nurse; 5 = Chief nurse
Job position	1 = Clinical nurse; 2 = Nursing teaching position; 3 = Nursing management position (head nurse, deputy head nurse, etc.); 4 = Nursing scientific research position
Employment nature	1 = Contract-based; 2 = Public institution; 3 = Other
Years of work experience	1 ≤ 2 years; 2 = 3–5 years; 3 = 6–10 years; 4 = 11–15 years; 5 = >15 years
Teaching experience	1 = Yes; 2 = No
Further education experience	1 = Yes; 2 = No
Specialist nurse role	1 = Yes; 2 = No
Self-directed learning competence	Original value input
General self-efficacy	Original value input

**Table 5:** Multivariate linear regression analysis of factors influencing core competence among infectious disease nurses (*n* = 392).

Variable	B	SE	*T*	*P*	B 95% CI		Collinearity diagnostics	
					Lower bound	Upper bound	Tolerance	VIF
(Constant)	29.095	7.974	3.649	< 0.001	13.416	44.775		
Hospital grade	2.374	1.012	2.347	0.019	0.385	4.363	0.781	1.281
Age group	−0.973	1.484	−0.656	0.513	−3.892	1.946	0.329	3.037
Marital status	1.287	2.188	0.588	0.557	−3.016	5.590	0.455	2.198
Fertility status	0.536	1.311	0.408	0.683	−2.043	3.114	0.469	2.131
Professional title	0.069	1.179	0.059	0.953	−2.249	2.387	0.425	2.353
Job position	3.660	1.398	2.617	0.009	0.910	6.410	0.717	1.395
Employment nature	−1.046	1.562	−0.670	0.503	−4.117	2.025	0.810	1.235
Years of work experience	2.436	0.865	2.816	0.005	0.735	4.137	0.325	3.077
Teaching experience	−6.207	1.690	−3.672	< 0.001	−9.530	−2.883	0.628	1.592
Further education experience	0.322	1.672	0.193	0.847	−2.965	3.609	0.751	1.331
Role of the specialist nurse	−3.034	1.576	−1.925	0.055	−6.133	0.065	0.785	1.274
Self-directed learning competence	0.660	0.042	15.567	< 0.001	0.577	0.743	0.384	2.607
General self-efficacy	0.349	0.159	2.190	0.029	0.036	0.663	0.380	2.630

*F* = 71.446, *p* < 0.001, *R* = 0.843, *R*^2^ = 0.711, adjusted *R*^2^ = 0.701.

## 4 Discussion

### 4.1 Analysis of the current core competence of infectious disease nurses

In this study, the mean total core competence score among infectious disease nurses was 133.72 ± 24.06, with an average item score of 3.93 ± 0.71, indicating that the overall core competence was above average. The differences in scores compared with previous study [e.g. Ma et al. (16)] may be influenced by variations in assessment instruments and scoring system. The current tool potentially provides a more accurate representation of the actual competence levels of infectious disease nurses.

Analysis of dimensional scores demonstrated that professional humanistic quality yielded the highest score (4.34 ± 0.71), indicating that participants generally possessed strong attributes in areas such as professional ethics, empathy, communication, and dedication. These competencies may be related to long-term work in specialized infectious disease settings, heightened societal expectations for care in this field, the emphasis placed on empathy and professional ethics in traditional nursing education, and the influence of team-based reinforcement of such qualities within clinical settings.

In contrast, professional development competence recorded the lowest mean score (3.43 ± 0.96), consistent with findings reported by Wu et al. (11). Several factors may contribute to this outcome. First, the high workload and risk associated with infectious disease care may limit opportunities for continued learning and scientific research. Second, current training systems may not adequately support the development of long-term professional growth, including scientific research competence, evidence-based practice, and innovation. Third, the absence of well-defined career development pathways and effective incentive mechanisms may hinder sustained development in specialized nursing roles.

Sustained high levels of clinical dedication in the absence of sufficient professional development opportunities may result in delayed knowledge updates, decline in care quality, accompanied by a heightened risk of job burnout (17). Recommended strategies to mitigate these risks include encouraging nurses to develop individualized career development plans and participate in quality improvement and scientific research projects; allocating protected time for continuing education and research; Establishment of structured career pathways, dedicated research funding, and ongoing access to educational resources may further enhance professional development among infectious disease nurses.

### 4.2 Self-directed learning competence among infectious disease nurses

The mean total score for self-directed learning competence among infectious disease nurses was 134.72 ± 25.35, with a mean item score of 3.96 ± 0.75, both higher than those reported in previous studies involving nurses specializing in diabetes care (18). These findings indicate that overall self-directed learning competence was above average. Among the four dimensions assessed, self-motivation belief recorded the highest mean score (4.02 ± 0.82), whereas self-evaluation yielded a relatively lower score (3.85 ± 0.76), consistent with previous findings reported by other scholars (19). This pattern indicates that infectious disease nurses possess strong motivation toward self-directed learning but experience challenges with self-evaluation, potentially due to a lack of objective criteria for self-assessment in clinical practice. To strengthen this capacity and overall self-directed learning, strategies such as maintaining learning notes, regularly analyzing learning outcomes, and identifying areas requiring improvement are recommended (20).

### 4.3 Influencing factors of core competence in infectious disease nurses

#### 4.3.1 Association between hospital grade and core competence

Nurses employed in higher-grade hospitals demonstrated higher levels of core competence compared to those working in lower-grade institutions (*p* = 0.019), consistent with findings from Bai et al. and Chen et al. (21, 22). Higher-grade hospitals often place greater emphasis on strengthening nurses' overall professional capabilities, provide more comprehensive training resources, and offer more structured educational opportunities. These hospitals also tend to facilitate greater access to continuing education and participation in large-scale infectious disease prevention and control initiatives. Additionally, promotion requirements in tertiary-level hospitals often emphasize competence development. To support competence enhancement in lower-grade hospitals, it is recommended to implement structured training systems, reinforce education in infectious disease prevention and emergency preparedness, facilitate opportunities for inter-institutional study visits in higher-grade hospitals, and develop regional nursing alliances to promote experience sharing and capacity building.

#### 4.3.2 Core competence among nurses in management, teaching, and scientific research roles

Nurses holding positions in nursing management, teaching, or scientific research demonstrated higher core competence levels than those in general clinical roles (*p* = 0.009), consistent with the analysis reported by Feng et al. in a study involving nurses specializing in pediatric critical care (22). Roles associated with management, education, or research typically require advanced competencies such as scientific inquiry, innovative thinking, critical thinking, and teaching competence (23). Nurses in these roles often receive greater access to training resources, more frequent opportunities for practical application, and clearer career development pathways, which may collectively contribute to higher competence levels. To strengthen overall competence among the nursing workforce, it is recommended that nursing managers provide increased training opportunities, implement mentorship programs, develop structured career pathways, and encourage nurses to pursue ongoing professional development.

#### 4.3.3 Years of clinical experience and teaching experience

Nurses with a longer duration of clinicals service demonstrated higher core competence (*p* = 0.005), consistent with previous findings (24, 25). Extended clinical exposure allows infectious disease nurses to refine essential clinical skills, strengthen problem-solving abilities, and develop greater psychological resilience when managing complex situations. Nurses with teaching experience also demonstrated higher core competence, consistent with findings reported by Liu et al. in a study of nurses specializing in stroke care (26). Clinical educators are generally required to meet formal entry standards and undergo competency assessments, which may encourage a greater emphasis on skill development (27). To further support these nurses, institutions are encouraged to provide platforms for professional development and create career advancement opportunities.

#### 4.3.4 Self-directed learning competence

Correlation analysis demonstrated a positive association between core competence and self-directed learning competence (*p* < 0.001), and multivariate regression analysis identified self-directed learning competence as an influencing factor. Infectious disease nurses must update their knowledge continuously due to the complexity and high-risk nature of their responsibilities, including emerging pathogens and evolving prevention and control strategies. Nurses with strong self-directed learning abilities can more effectively acquire new knowledge and develop clinical skills, thereby responding more efficiently to clinical challenges (28). Furthermore, self-directed learning is closely related to professional self-identity and post competency, indicating that enhancing self-directed learning may indirectly strengthen core competence. Recommended strategies include implementing diversified teaching models, linking self-directed learning outcomes with performance evaluations, and creating supportive work environments with protected learning time.

#### 4.3.5 General self-efficacy

The correlation analysis demonstrated a positive association between core competence and self-efficacy (*p* < 0.001), with regression analysis confirming self-efficacy as an influencing factor, consistent with findings from a prior studies involving newly graduated nurses and those working in emergency departments (29, 30). Nurses with higher self-efficacy are more capable of managing the complex demands of infectious disease care and more motivated to acquire new knowledge and skills. However, due to the intense workload and elevated occupational risks, infectious disease nurses may be susceptible to job burnout and reduced self-efficacy. To address this, evidence-based strategies are recommended, including case-based training to strengthen problem-solving abilities, building a supportive team environment, providing access to psychological counseling services, and offering stress management interventions to bolster mental resilience (31). Additionally, optimizing work allocation and minimizing non-essential tasks may further support self-efficacy.

## 5 Conclusion

This study assessed core competence among 392 infectious disease nurses and found that overall core competence levels were above average. Multivariate analysis identified hospital grade, job position, years of clinical experience, teaching experience, self-directed learning competence, and self-efficacy as influencing factors. These findings suggest that nursing managers may strengthen the development of infectious disease nursing teams by improving training systems, establishing clear career development pathways, and enhancing nurses' self-confidence and professional self-identity through psychological guidance and career planning initiatives. Targeted strategies aimed at improving self-directed learning competence and self-efficacy may further enhance core competence, thereby supporting high-quality care for patients with infectious diseases and contributing to public health objectives. This study employed a convenience sampling method due to time constraints and considerations of feasibility in data collection. The survey was facilitated through existing professional contacts with infectious disease departments in selected hospitals, enabling efficient data collection within a limited timeframe. However, this method may introduce sampling bias and limit the generalizability of the findings. Future research should consider the use of stratified sampling or other probabilistic methods to reduce bias. Additionally, further investigation into the effects of occupational stressors, such as workload and job burnout, on core competence is warranted to provide a more comprehensive evidence base for interventions aimed at strengthening core competence among infectious disease nurses.

## Data Availability

The original contributions presented in the study are included in the article/Supplementary material, further inquiries can be directed to the corresponding author.
