# Supplementary material for: Influence of self-efficacy and self-directed learning competence on core competence among nurses specializing in infectious diseases: a cross-sectional study
Source: Front Public Health. 2026 May 22;14:1775251. doi: 10.3389/fpubh.2026.1775251 (PMC13236676; doi:10.3389/fpubh.2026.1775251)
Supplement: Supplementary file 1 [file Data_Sheet_1.docx]

**Survey on Core Competence of Infectious Disease Nurses**

Dear Nursing Colleague,

Hello! I am a nurse from Shanxi Bethune Hospital, and I am inviting you to participate in a study on the core competence of nurses working in infectious disease departments. Your support would be greatly appreciated.

This questionnaire consists of four parts:

1. General Information Questionnaire
2. Core Competence Questionnaire for Infectious Disease Specialist Nurses
3. Self-Directed Learning Ability Questionnaire
4. Self-Efficacy Scale

The survey is anonymous and will be used solely for research purposes. Please complete the questionnaire based on your actual situation. The information you provide will serve as valuable data for our study, and we guarantee that all responses will be kept strictly confidential.

Thank you very much for taking the time out of your busy schedule to participate in this survey. We sincerely appreciate your support!

**Part 1: General Information Questionnaire**

1. Name of your hospital [Open-ended]
2. Hospital level [Single choice]

A. Secondary Grade A

B. Secondary Grade B

C. Tertiary Grade A

D. Tertiary Grade B

E. Other

1. Hospital type [Single choice]

A. General hospital

B. Specialized hospital

1. Age group [Single choice]

A. 21–30 years

B. 31–40 years

C. 41–50 years

D. >51 years

1. Marital status [Single choice]

A. Unmarried

B. Married

C. Divorced

D. Widowed

E. Other

1. Parental status [Single choice]

A. None

B. One child

C. Two or more children

1. Highest educational level [Single choice]

A. Secondary technical school

B. Junior college (associate degree)

C. Bachelor’s degree

D. Master’s degree or above

1. Professional title [Single choice]

A. Nurse

B. Nurse practitioner (Junior)

C. Nurse-in-charge (Intermediate)

D. Associate chief nurse

E. Chief nurse

1. Job position [Single choice]

A. Clinical nurse

B. Nursing education

C. Nursing management (e.g., head nurse, deputy head nurse)

D. Nursing research

1. Employment type [Single choice]

A. Contract-based

B. Government-funded (established post)

C. Other

1. Years of experience in infectious disease-related work (For less than one year, round to the nearest whole year; e.g., 2 years 2 months = 2 years; 2 years 7 months = 3 years) [Single choice]

A. ≤2 years

B. 3–5 years

C. 6–10 years

D. 11–15 years

E. >15 years

1. Do you have teaching experience? [Single choice]

A. Yes

B. No

1. Do you have experience of external training or professional development (e.g., visiting scholar/training programs)? [Single choice]

A. Yes

B. No

**Part 2: Core Competence Questionnaire for Infectious Disease Specialist Nurses**

Please rate each item based on your actual situation. Each item is scored from 1 (strongly disagree) to 5 (strongly agree).

**Professional Development Competence**

(Matrix single-choice)

| **Item** | **Strongly Disagree** | **Disagree** | **Slightly Agree** | **Agree** | **Strongly Agree** |
| --- | --- | --- | --- | --- | --- |
| 1. Ability to deliver teaching sessions on infectious diseases | ○ | ○ | ○ | ○ | ○ |
| 2. Ability to allocate, supervise, guide, and manage the work of infectious disease nurses | ○ | ○ | ○ | ○ | ○ |
| 3. Ability to train general nurses during infectious disease emergencies | ○ | ○ | ○ | ○ | ○ |
| 4. Possess innovation awareness and the ability to improve and optimize infectious disease nursing processes and protective equipment | ○ | ○ | ○ | ○ | ○ |
| 5. Ability to collaborate with other departments and effectively coordinate human and material resources | ○ | ○ | ○ | ○ | ○ |
| 6. Ability to provide clinical teaching in infectious disease nursing | ○ | ○ | ○ | ○ | ○ |
| 7. Ability to evaluate and improve the quality of infectious disease nursing interventions | ○ | ○ | ○ | ○ | ○ |
| 8. Ability to search and retrieve literature through multiple sources and critically evaluate its quality | ○ | ○ | ○ | ○ | ○ |
| 9. Ability to manage materials, including medications, consumables, documents, and equipment in infectious disease departments | ○ | ○ | ○ | ○ | ○ |
| 10. Ability to design research topics related to infectious disease nursing | ○ | ○ | ○ | ○ | ○ |
| 11. Ability to write academic papers | ○ | ○ | ○ | ○ | ○ |

**Infection Prevention and Control Competence**

(Matrix single-choice)

| **Item** | **Strongly Disagree** | **Disagree** | **Slightly Agree** | **Agree** | **Strongly Agree** |
| --- | --- | --- | --- | --- | --- |
| 12. Ability to properly handle medical waste generated by patients with infectious diseases (e.g., infectious diarrhea, HIV/AIDS, COVID-19) | ○ | ○ | ○ | ○ | ○ |
| 13. Mastery of procedures for donning and using personal protective equipment | ○ | ○ | ○ | ○ | ○ |
| 14. Clear understanding of protection requirements for different infectious diseases | ○ | ○ | ○ | ○ | ○ |
| 15. Ability to manage occupational exposure risks such as skin/mucosal exposure and sharps injuries | ○ | ○ | ○ | ○ | ○ |
| 16. Mastery of disinfection methods for infectious disease wards and equipment | ○ | ○ | ○ | ○ | ○ |
| 17. Understanding of different isolation types (airborne, contact, droplet isolation) | ○ | ○ | ○ | ○ | ○ |
| 18. Mastery of common isolation techniques and methods (e.g., patient flow isolation, hospital layout-based isolation, inpatient isolation) | ○ | ○ | ○ | ○ | ○ |
| 19. Understanding of standard precautions and transmission-based precautions | ○ | ○ | ○ | ○ | ○ |
| 20. Familiarity with commonly used physical and chemical disinfection methods | ○ | ○ | ○ | ○ | ○ |

**Infectious Disease Nursing Competence**

(Matrix single-choice)

| **Item** | **Strongly Disagree** | **Disagree** | **Slightly Agree** | **Agree** | **Strongly Agree** |
| --- | --- | --- | --- | --- | --- |
| 21. Knowledge of pathogenesis, epidemiological history, nursing priorities, and health education for common infectious diseases | ○ | ○ | ○ | ○ | ○ |
| 22. Familiarity with commonly used medications for infectious diseases, including administration principles, routes, side effects, and precautions | ○ | ○ | ○ | ○ | ○ |
| 23. Mastery of emergency nursing techniques for critically ill infectious disease patients | ○ | ○ | ○ | ○ | ○ |
| 24. Familiarity with nursing care for common symptoms such as fever, rash, diarrhea, seizures, and convulsions | ○ | ○ | ○ | ○ | ○ |
| 25. Ability to develop individualized nursing care plans based on patients’ conditions | ○ | ○ | ○ | ○ | ○ |
| 26. Familiarity with nursing care for common procedures in infectious disease departments (e.g., Sengstaken–Blakemore tube compression hemostasis, lactulose enema, invasive arterial blood pressure monitoring) | ○ | ○ | ○ | ○ | ○ |

**Professional Humanistic Qualities**

(Matrix single-choice)

| **Item** | **Strongly Disagree** | **Disagree** | **Slightly Agree** | **Agree** | **Strongly Agree** |
| --- | --- | --- | --- | --- | --- |
| 27. Respect patients in nursing practice, demonstrate zero discrimination toward infectious disease patients, and protect patient privacy | ○ | ○ | ○ | ○ | ○ |
| 28. Ability to provide psychological support and mental health care for infectious disease patients | ○ | ○ | ○ | ○ | ○ |
| 29. Ability to provide health education to patients and the public | ○ | ○ | ○ | ○ | ○ |
| 30. Ability to self-regulate psychologically and manage stress in infectious disease nursing work | ○ | ○ | ○ | ○ | ○ |
| 31. Passion for infectious disease nursing and a strong sense of professional identity | ○ | ○ | ○ | ○ | ○ |

**Emergency Response Competence for Infectious Disease Outbreaks**

(Matrix single-choice)

| **Item** | **Strongly Disagree** | **Disagree** | **Slightly Agree** | **Agree** | **Strongly Agree** |
| --- | --- | --- | --- | --- | --- |
| 32. Regular participation in emergency drills for infectious disease outbreaks | ○ | ○ | ○ | ○ | ○ |
| 33. Familiarity with response procedures for public health emergencies related to infectious diseases | ○ | ○ | ○ | ○ | ○ |
| 34. Ability to anticipate and identify infectious disease outbreaks | ○ | ○ | ○ | ○ | ○ |

**Part 3: Self-Directed Learning Ability Questionnaire for Nurses**

Please rate each item based on your actual situation. Each item is scored from 1 (strongly disagree) to 5 (strongly agree).

**Self-Motivation Beliefs**

(Matrix single-choice)

| **Item** | **Strongly Disagree** | **Disagree** | **Slightly Agree** | **Agree** | **Strongly Agree** |
| --- | --- | --- | --- | --- | --- |
| 1. I am confident that I have strong comprehension abilities | ○ | ○ | ○ | ○ | ○ |
| 2. Regardless of my learning outcomes, I never doubt my abilities | ○ | ○ | ○ | ○ | ○ |
| 3. I believe I am capable of solving problems encountered during learning | ○ | ○ | ○ | ○ | ○ |
| 4. I believe I can apply what I have learned to my work | ○ | ○ | ○ | ○ | ○ |
| 5. I think learning is an interesting activity | ○ | ○ | ○ | ○ | ○ |
| 6. I am willing to participate in various learning activities organized by the hospital (e.g., professional training, lectures) | ○ | ○ | ○ | ○ | ○ |
| 7. When new equipment, technologies, or methods are introduced in the ward, I am eager to understand them | ○ | ○ | ○ | ○ | ○ |
| 8. I take the initiative to learn the knowledge and skills I need | ○ | ○ | ○ | ○ | ○ |
| 9. Once I decide to learn something, I will make time for it regardless of how busy I am | ○ | ○ | ○ | ○ | ○ |
| 10. When I encounter questions at work, I always try to find the answers | ○ | ○ | ○ | ○ | ○ |
| 11. I consider learning new knowledge to be a meaningful challenge | ○ | ○ | ○ | ○ | ○ |
| 12. I often choose to read journals or books that help me better understand nursing practice, even if it requires considerable time and effort | ○ | ○ | ○ | ○ | ○ |
| 13. I feel a sense of satisfaction after mastering certain knowledge | ○ | ○ | ○ | ○ | ○ |
| 14. Learning new knowledge is helpful for my work | ○ | ○ | ○ | ○ | ○ |

**Task Analysis**

(Matrix single-choice)

| **Item** | **Strongly Disagree** | **Disagree** | **Slightly Agree** | **Agree** | **Strongly Agree** |
| --- | --- | --- | --- | --- | --- |
| 15. Before learning, I can identify my learning needs and set learning goals | ○ | ○ | ○ | ○ | ○ |
| 16. I usually set specific learning goals | ○ | ○ | ○ | ○ | ○ |
| 17. While setting long-term goals, I also establish short-term goals | ○ | ○ | ○ | ○ | ○ |
| 18. I generally set high-level goals that are achievable with effort | ○ | ○ | ○ | ○ | ○ |
| 19. I am familiar with commonly used learning methods | ○ | ○ | ○ | ○ | ○ |
| 20. I can select appropriate and effective learning strategies based on my situation | ○ | ○ | ○ | ○ | ○ |

**Self-Monitoring and Regulation**

(Matrix single-choice)

| **Item** | **Strongly Disagree** | **Disagree** | **Slightly Agree** | **Agree** | **Strongly Agree** |
| --- | --- | --- | --- | --- | --- |
| 21. I develop practical learning plans based on my work situation | ○ | ○ | ○ | ○ | ○ |
| 22. When encountering difficulties in learning, I seek help from others | ○ | ○ | ○ | ○ | ○ |
| 23. I am familiar with ways to access various information resources (e.g., nursing journals, databases, internet) | ○ | ○ | ○ | ○ | ○ |
| 24. I can effectively use different methods to obtain the learning materials I need | ○ | ○ | ○ | ○ | ○ |
| 25. I can analyze, synthesize, and organize collected information into valuable reports | ○ | ○ | ○ | ○ | ○ |
| 26. I can persist in learning despite difficulties | ○ | ○ | ○ | ○ | ○ |
| 27. When I intend to study, I can quickly enter a focused learning state | ○ | ○ | ○ | ○ | ○ |
| 28. I can monitor my learning progress and identify problems in a timely manner | ○ | ○ | ○ | ○ | ○ |
| 29. I can adjust my learning methods when they are ineffective | ○ | ○ | ○ | ○ | ○ |
| 30. I can adjust my learning plans according to actual circumstances | ○ | ○ | ○ | ○ | ○ |

**Self-Evaluation**

(Matrix single-choice)

| **Item** | **Strongly Disagree** | **Disagree** | **Slightly Agree** | **Agree** | **Strongly Agree** |
| --- | --- | --- | --- | --- | --- |
| 31. After completing a learning activity, I mentally review the entire process | ○ | ○ | ○ | ○ | ○ |
| 32. After learning, I often summarize experiences and lessons learned | ○ | ○ | ○ | ○ | ○ |
| 33. I clearly understand how this learning experience can guide future learning | ○ | ○ | ○ | ○ | ○ |
| 34. Poor learning outcomes are due to my limited ability | ○ | ○ | ○ | ○ | ○ |

**Part 4: General Self-Efficacy Scale**Please rate each item based on your actual situation. Each item is scored from 1 (Not at all true) to 4 (Exactly true).

**General Self-Efficacy Scale**

(Matrix single-choice)

| **Item** | **Not at all true** | **Hardly true** | **Moderately true** | **Exactly true** |
| --- | --- | --- | --- | --- |
| 1. If I try my best, I can always solve the problem | ○ | ○ | ○ | ○ |
| 2. Even if someone opposes me, I can still find ways to achieve what I want | ○ | ○ | ○ | ○ |
| 3. For me, sticking to my goals and achieving them is easy | ○ | ○ | ○ | ○ |
| 4. I am confident that I can effectively deal with unexpected situations | ○ | ○ | ○ | ○ |
| 5. With my intelligence, I can handle unexpected situations | ○ | ○ | ○ | ○ |
| 6. If I put in the necessary effort, I can solve most problems | ○ | ○ | ○ | ○ |
| 7. I can remain calm when facing difficulties because I trust my problem-solving abilities | ○ | ○ | ○ | ○ |
| 8. When facing a difficult problem, I can usually find several solutions | ○ | ○ | ○ | ○ |
| 9. When I am in trouble, I can usually think of ways to deal with it | ○ | ○ | ○ | ○ |
| 10. No matter what happens to me, I can handle it well | ○ | ○ | ○ | ○ |
